# ApoA-I mimetic administration, but not increased apoA-I-containing HDL, inhibits tumour growth in a mouse model of inherited breast cancer

**DOI:** 10.1038/srep36387

**Published:** 2016-11-03

**Authors:** Lídia Cedó, Annabel García-León, Lucía Baila-Rueda, David Santos, Victor Grijalva, Melanie Raquel Martínez-Cignoni, José M. Carbó, Jari Metso, Laura López-Vilaró, Antonio Zorzano, Annabel F. Valledor, Ana Cenarro, Matti Jauhiainen, Enrique Lerma, Alan M. Fogelman, Srinivasa T. Reddy, Joan Carles Escolà-Gil, Francisco Blanco-Vaca

**Affiliations:** 1 Institut d’Investigacions Biomèdiques (IIB) Sant Pau, Barcelona, Spain; 2 CIBER de Diabetes y Enfermedades Metabólicas Asociadas, CIBERDEM, Barcelona, Spain; 3 Unidad Clínica y de Investigación en Lípidos y Arteriosclerosis, Hospital Universitario Miguel Servet, Instituto de Investigación Sanitaria Aragón (IIS Aragón), Zaragoza, Spain; 4 Department of Medicine, University of California, Los Angeles, CA, USA; 5 Departament de Bioquímica i Biologia Molecular, Universitat Autònoma de Barcelona, Barcelona, Spain; 6 Nuclear Receptor Group, Department of Cell Biology, Physiology and Immunology, School of Biology, University of Barcelona, Barcelona, Spain; 7 National Institute for Health and Welfare, Genomics and Biomarkers Unit, and Minerva Foundation Institute for Medical Research, Biomedicum, Helsinki, Finland; 8 Departament de Patologia, Hospital de la Santa Creu i Sant Pau, Barcelona, Spain; 9 Institute for Research in Biomedicine (IRB Barcelona), Barcelona, Spain; 10 Departament de Ciències Morfològiques, Universitat Autònoma de Barcelona, Barcelona, Spain

## Abstract

Low levels of high-density lipoprotein cholesterol (HDLc) have been associated with breast cancer risk, but several epidemiologic studies have reported contradictory results with regard to the relationship between apolipoprotein (apo) A-I and breast cancer. We aimed to determine the effects of human apoA-I overexpression and administration of specific apoA-I mimetic peptide (D-4F) on tumour progression by using mammary tumour virus-polyoma middle T-antigen transgenic (PyMT) mice as a model of inherited breast cancer. Expression of human apoA-I in the mice did not affect tumour onset and growth in PyMT transgenic mice, despite an increase in the HDLc level. In contrast, D-4F treatment significantly increased tumour latency and inhibited the development of tumours. The effects of D-4F on tumour development were independent of 27-hydroxycholesterol. However, D-4F treatment reduced the plasma oxidized low-density lipoprotein (oxLDL) levels in mice and prevented oxLDL-mediated proliferative response in human breast adenocarcinoma MCF-7 cells. In conclusion, our study shows that D-4F, but not apoA-I-containing HDL, hinders tumour growth in mice with inherited breast cancer in association with a higher protection against LDL oxidative modification.

Breast cancer is the most commonly diagnosed cancer and the second most common cause of cancer-related death among women^1^. Malignant proliferation of breast cancer tissue has been associated with changes in plasma lipid and lipoprotein levels. Low levels of high-density lipoprotein cholesterol (HDLc) have been associated with breast cancer risk^2^,^3^,^4^. However, the relationship between apolipoprotein (apo) A-I levels and breast cancer risk is not clear, as results of published studies are contradictory^5^,^6^.

HDL is an heterogenous mixture of subpopulations of HDL particles ranging from nascent discoidal to mature spherical particles, which play an important role in reverse cholesterol transport from peripheral cells to the liver for biliary excretion^7^. The majority of HDL particles contain either a single copy or multiple copies of apoA-I. ApoA-I plays a role in promoting cholesterol release from cells; possesses anti-inflammatory, antioxidant and anti-apoptotic properties; and also influences innate immunity^8^.

As the lipoprotein metabolism, inflammation and cancer have been reported to be inter-related^9^, the anti-tumourigenic role of apoA-I is currently under investigation. Some studies have demonstrated that human apoA-I-containing HDL has potent anti-tumour activity in xenograft mouse models of ovarian cancer and mouse models of malignant melanoma and Lewis lung carcinoma^10^,^11^. In one study, a synthetic reconstituted HDL was also found to inhibit B cell lymphoma xenografts in mice, although these effects were not observed after native human HDL was administered^12^.

ApoA-I mimetic peptides, which mimic the distribution of the charge and structure of portions of apoA-I, are considered as potential therapeutic agents for preventing a variety of inflammation-related diseases, including cancer (reviewed in ref. 13). In particular, the peptide 4F contains 18 amino acids that form an amphipathic α-helix, which allows the peptide to mimic the antioxidant and anti-inflammatory properties of apoA-I, but with much higher potency^14^. D-4F is synthesized from D-amino acids and L-4F from L-amino acids, and both peptides have been shown to have similar *in vitro* and *in vivo* properties, which indicates that the stereo-specificity of the peptide is not related to its activity^15^. Two reports have demonstrated that both D-4F and L-4F significantly reduce tumour growth in xenograft mouse models of ovarian and colon cancer, and this effect is partly brought about by their binding to and removing lysophosphatidic acids^10^,^16^. Recent data have also shown that internalization of oxidized low-density lipoprotein (oxLDL) by mammary epithelial cells may play a significant role in breast cancer by triggering a variety of proliferative and pro-inflammatory mechanisms^17^. However, the potential *in vivo* therapeutic effect of apoA-I or its mimetics on breast cancer has not been studied.

In the present study, mammary tumour virus-polyoma middle T-antigen transgenic (PyMT) mice, which spontaneously develop widespread multifocal adenocarcinomas in the mammary gland, were backcrossed with human apoA-I transgenic mice or administered D-4F, and tumour onset and growth were analyzed.

## Results

### Overexpression of human apoA-I does not hinder tumour development in PyMT mice

**Figure 1:**
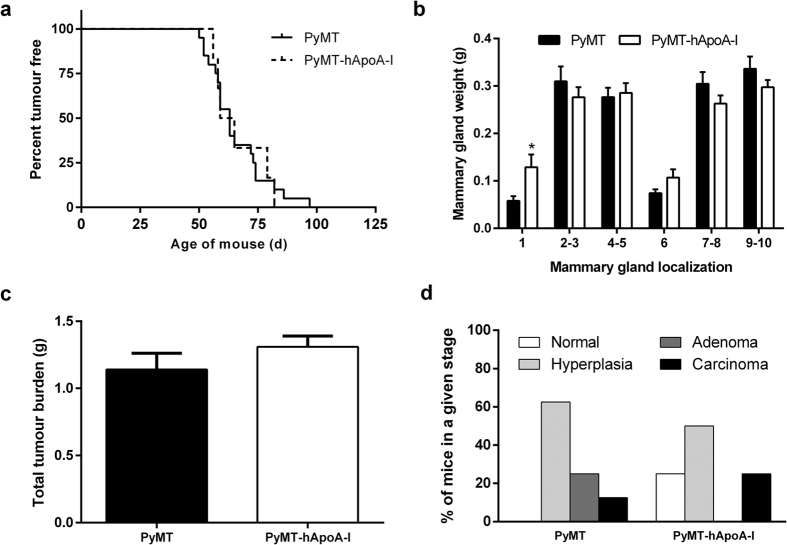
Effects of hApoA-I overexpression on tumour development in PyMT mice. (**a**) Tumour latency in PyMT and PyMT-hApoA-I mice. (**b**) Mammary gland weight at the end of the study. (**c**) Total tumour burden in PyMT and PyMT-hApoA-I mice. (**d**) Each section from right inguinal mammary gland was graded according to the maximum lesion grade and classified as follows: normal, hyperplasia, adenoma and carcinoma. The percentage of mice in each histopathologic stage is shown. In (**b**,**c**), the values shown represent the mean ± SEM for 10 individual animals per group, and *indicates p ≤ 0.05 vs. the PyMT group.

Female PyMT mice and those expressing human apoA-I (PyMT-hApoA-I) were examined for palpable tumours; the first tumours were detected within 50 days. However, tumour latency was not affected by hApoA-I expression: T_50_, defined as the time in which 50% of the animals were free of tumour, was 63 days in PyMT mice and 62 days in PyMT-hApoA-I mice (Fig. 1a). Mice were euthanized at 19 weeks of age, and the effect of hApoA-I expression on tumour burden was assessed. Mammary gland weight was not modified by hApoA-I expression in most mammary gland localizations, except in one instance where tumour weight was significantly greater (Fig. 1b). Total tumour burden was also not affected (Fig. 1c), and neither was total body weight (Table 1). Longitudinal sections of the right inguinal mammary gland were prepared, and the maximum lesion grade in each animal was assessed. The lesion grade profile was similar between the two groups, as the percentage of adenomatous and carcinomatous tissue was similar in both groups (Fig. 1d). Representative histological images are shown in Supplementary Figure S1.

### Tumour development is inhibited in PyMT mice treated with D-4F

**Figure 2:**
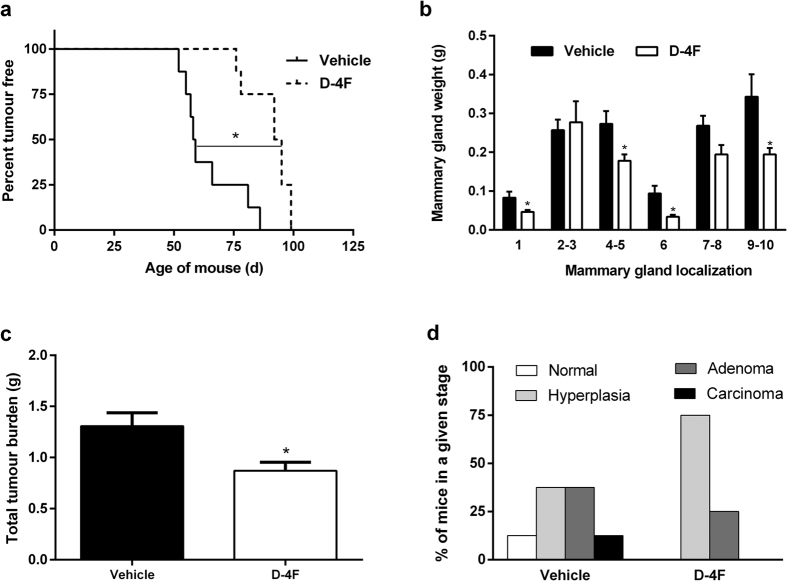
Effects of D-4F treatment on tumour development in PyMT mice. (**a**) Tumour latency in PyMT mice treated with 10 mg/kg of D-4F or vehicle; significant (p ≤ 0.05) differences between curves are indicated by a connecting solid line and an asterisk. (**b**) Mammary gland weight at the end of the study. Two-way ANOVA showed that D-4F treatment reduced mammary gland weight without interaction between mammary gland localization and treatment. (**c**) Total tumour burden in PyMT mice treated with D-4F or vehicle. (**d**) Each section from right inguinal mammary gland was graded according to the maximum lesion grade and classified as follows: normal, hyperplasia, adenoma or carcinoma. The percentage of mice in each histopathologic stage is shown. In (**b,c**), the values shown represent the mean ± SEM for 8 individual animals per group, and *indicates p ≤ 0.05 vs. the vehicle group.

To determine whether the apoA-I mimetic peptide D-4F could inhibit tumour development, D-4F or a vehicle was administered daily until the first tumours were detectable, and, then, they were administered three times per week until the mice reached 19 weeks of age. Tumour latency was significantly increased in the mice treated with D-4F (Fig. 2a), with T_50_ being 93.5 days in the D-4F-treated PyMT mice and 58.5 days in the vehicle-treated PyMT mice. Mammary gland weight was significantly lower in cervical mammary glands 1 and 6 and in inguinal mammary glands 4–5 and 9–10 (Fig. 2b). Similarly, total tumour burden was also significantly decreased in D-4F-treated mice (Fig. 2c), which caused a reduction in body weight (Table 2). Moreover, histopathologic examination showed that D-4F treatment resulted in an increase in the percentage of mice in the normal or hyperplasia stage and a decrease in the percentage of mice with adenoma and carcinoma lesions (Fig. 2d).

### Human apoA-I and D-4F have divergent effects on HDLc, preβ-HDL and 27-hydroxycholesterol levels

As expected^18^, the PyMT-hApoA-I mice had higher levels of total serum cholesterol, which was mainly caused by an increase in the HDL mass, mainly human apoA-I and cholesterol (Table 1). In contrast, D-4F administration resulted in a decrease in the HDLc levels without altering the mouse apoA-I levels (Table 2). As expected^19^,^20^, the pre-β HDL levels were higher in the D-4F-treated mice than in the vehicle-treated mice (Table 2).

**Figure 3:**
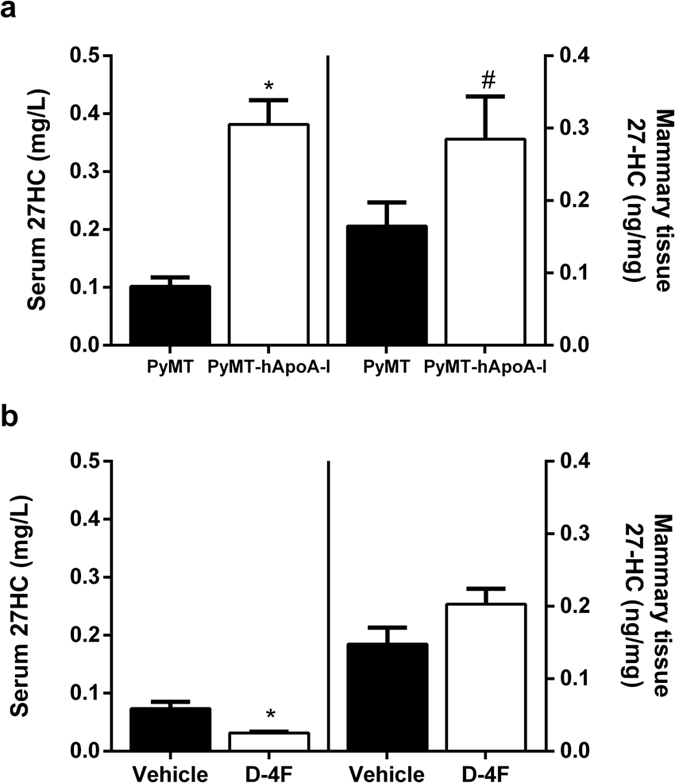
Effects of hApoA-I overexpression or D-4F treatment on 27-HC levels in PyMT mice. The 27-HC levels in serum and left thoracic mammary tissue in (**a**) PyMT and PyMT-hApoA-I mice and in (**b**) PyMT mice treated with 10 mg/kg of D-4F or the vehicle. The values shown are the mean ± SEM for 5-8 individual animals per group. *indicates p ≤ 0.05 and ^#^p ≤ 0.1 vs. PyMT or the vehicle group, respectively.

**Figure 4:**
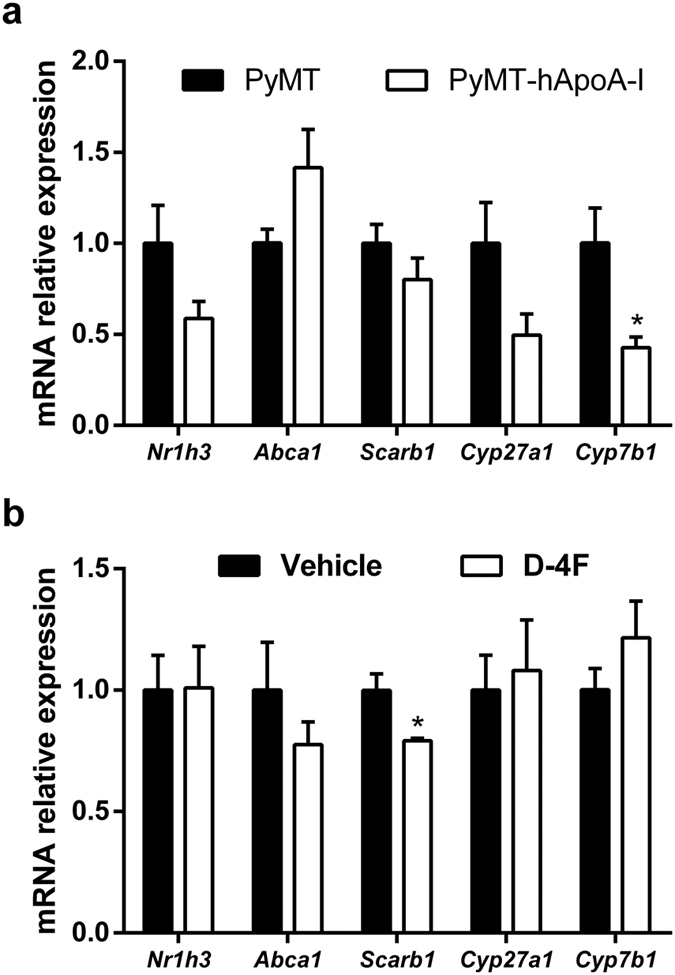
Effects of hApoA-I overexpression or D-4F treatment on mammary gland gene expression in PyMT mice. Right cervical mammary gland gene expression in (**a**) PyMT and PyMT-hApoA-I mice and in (**b**) PyMT mice treated with 10 mg/kg of D-4F or vehicle, at the end of the study. The signal in the PyMT and vehicle group was set at a normalized value of 1 arbitrary unit (AU). *Gapdh* was used as the internal control. The values represent the mean ± SEM for 5-7 individual animals per group, and *indicates p ≤ 0.05 vs. PyMT or the vehicle group, respectively.

Since 27-hydroxycholesterol (27-HC) has been found to promote breast cancer *in vivo*^21^,^22^, we analyzed the 27-HC levels in serum and in mammary tissue. 27-HC was higher both in the serum and mammary tissue of PyMT-hApoA-I mice than the other mice (Fig. 3a). However, the expression of genes involved in HDL-mediated cholesterol efflux and influx were unchanged in their mammary tissue (Fig. 4a). Furthermore, the expression of *Cyp7b1*, the main enzyme responsible for the catabolism of 27-HC, was found to be downregulated in the mammary tissue of PyMT-hApoA-I mice (Fig. 4a). Conversely, D-4F reduced the serum 27-HC levels (Fig. 3b), but it did not affect the expression of either *Cyp7b1* or *Cyp27a1*, the enzyme responsible for the generation of 27-HC from cholesterol (Fig. 4b). Consistent with this latter finding, the mammary gland 27-HC levels were not affected by D-4F (Fig. 3b). D-4F administration reduced *Scarb1* gene expression in mammary tissue (Fig. 4b). However, the SR-BI protein levels were not significantly altered (0.51 ± 0.06 vs. 1.00 ± 0.47 arbitrary units (AU) in D-4F- and vehicle-treated mice respectively; p = 0.49).

### The effects of D-4F on tumour development are independent of the pro-inflammatory phospholipids and prototypic markers of macrophage activation

Since previous reports found that D-4F considerably reduced pro-inflammatory phospholipids in a mouse model of ovarian cancer^15^, we also evaluated the serum lysophosphatidic acid (LPA) levels. However, D-4F did not affect the LPA levels in PyMT mice, which were also unchanged by hApoA-I expression (Supplementary Fig. S2). We further analyzed the expression of genes associated with either pro-inflammatory or alternative macrophage activation in the mammary glands of mice, but neither hApoA-I nor D-4F influenced the M1/M2 profile (Supplementary Fig. S3).

### D-4F reduces serum oxLDL levels in PyMT mice and hinders oxLDL-mediated proliferate response in human breast adenocarcinoma cells

**Figure 5:**
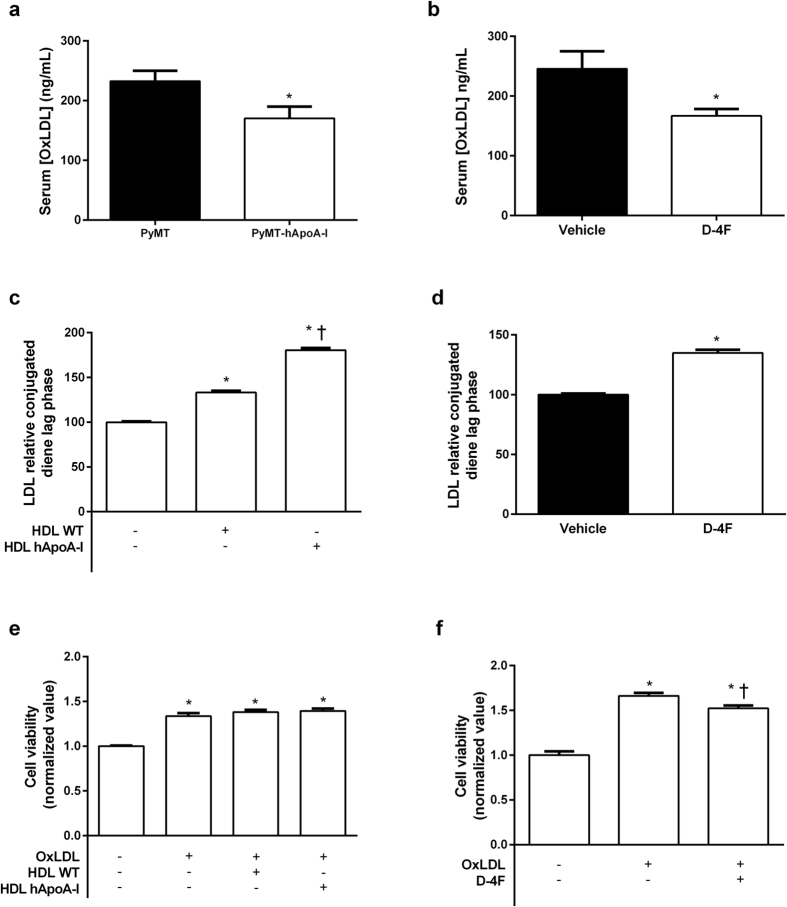
Effects of hApoA-I or D-4F on the serum oxLDL levels in PyMT mice and oxLDL-mediated proliferative response in MCF-7 cells. (**a**) Serum oxLDL levels in PyMT and PyMT-hApoA-I mice. The values represent the mean ± SEM for 8-10 individual animals. *Indicates p ≤ 0.05 vs. PyMT. (**b**) Serum oxLDL levels in PyMT mice treated with D-4F or the vehicle. The values represent the mean ± SEM for 4–5 individual animals per group, and *indicates p ≤ 0.05 vs. the vehicle. (**c**) Activity of HDL isolated from wild-type (WT) or hApoA-I mice against LDL oxidative modification. Results are expressed as the lag phase of conjugated diene formation kinetics, which is presented as the relative lag phase of LDL oxidized without HDL. The values represent the mean ± SEM for 5 replicates per group. *Indicates p ≤ 0.05 vs. LDL oxidized without HDL, and ^†^indicates p ≤ 0.05 vs. LDL oxidized with HDL from WT. (**d**) Activity of D-4F against LDL oxidative modification. The results are expressed as the lag phase of conjugated diene formation kinetics, which is presented as the relative lag phase of LDL oxidized without D-4F. The values represent the mean ± SEM for 5 replicates per group. *indicates p ≤ 0.05 vs. LDL oxidized without D-4F. (**e**) Cell viability of MCF-7 cells treated with 100 mg/L of human oxLDL and human oxLDL combined with 100 mg/L of HDL isolated from WT or hApoA-I mice. The signal in the control group was set at a normalized value of 1 AU. The values represent the mean ± SEM for replicates per group and, *indicates p ≤ 0.05 vs. untreated cells. (**f**) Cell viability of MCF-7 cells treated with 100 mg/L of human oxLDL with or without 20 mg/L of D-4F. The signal of the control group was set at a normalized value of 1 AU. The values represent the mean ± SEM for 12 replicates per group. *indicates p ≤ 0.05 vs. untreated cells, and ^†^indicates p ≤ 0.05 vs. cells treated with oxLDL alone.

Overexpression of hApoA-I reduced oxLDL levels in PyMT mice (Fig. 5a), but these changes were not correlated with total tumour burden (r = −0.22, p = 0.36). D-4F also reduced serum oxLDL levels in PyMT mice (Fig. 5b), but this was correlated with total tumour burden (r = 0.71, p = 0.03). Both hApoA-I overexpression (Fig. 5c) and D-4F treatment (Fig. 5d) induced a longer lag phase in copper-induced LDL oxidation, indicating that extensive oxidation of LDL was delayed. OxLDL increases the viability of the human mammary adenocarcinoma cell line MCF-7, but while hApoA-I-containing HDL was not able to counteract the effect of oxLDL (Fig. 5e), D-4F hindered in part the oxLDL-mediated proliferative response (Fig. 5f). When MCF-7 cells were treated with D-4F alone, it also reduced cell viability without affecting their migration capacity in the wound healing process or the expression of the genes involved in the cholesterol efflux and influx processes (Supplementary Fig. S4). This change was also associated with improved ability of D-4F to stimulate cholesterol efflux from the MCF-7 cells (Supplementary Fig. S4c).

## Discussion

Recent publications have emphasized the importance of lipids, mainly cholesterol, in cancer progression^22^,^23^,^24^,^25^,^26^, whereas the main HDL protein, apoA-I, has been considered anti-tumourigenic^10^,^11^. Mouse apoA-I and human apoA-I have 65% sequence homology, and human apoA-I is functionally more potent than mouse apoA-I^7^. Therefore, the present study was designed to examine the effect of hApoA-I overexpression and administration of the apoA-I mimetic peptide D-4F on breast cancer development. To the best of our knowledge, these are the first set of results to demonstrate that D-4F increased tumour latency and inhibited tumour development in a mouse model of inherited breast cancer. These effects were closely associated with the ability of D4-F to prevent oxLDL formation. Several lines of evidence support the role of oxLDL in cancer development. For example, one study showed that the serum oxLDL levels are associated with an increased risk of breast cancer^27^. Furthermore, oxLDL was reported to trigger proliferative and pro-inflammatory signalling in MCF10A cells partly by stimulation of MiR-21^17^. In addition, oxLDL (lectin-like) receptor 1 (OLR1), the main receptor for internalization of oxLDL, has been reported to play a major role in tumour growth in xenograft mouse models of breast cancer and is critical for cell transformation^9^,^28^,^29^. Consistent with these observations, we found that MCF-7 exhibited an increased proliferative response to oxLDL and, also, that D-4F was able to reduce oxLDL-mediated MCF-7 viability. It is possible that the *in vitro* inhibitory effects of D-4F against oxLDL formation were lower than its *in vivo* effects because LDL was already in an oxidized state when it was added to the cultured cells. Indeed, D-4F significantly reduced the serum oxLDL levels, which were highly correlated with the total tumour burden; thus, the inhibition of cell growth could be one of the mechanisms underlying the apoA-I mimetic action of D-4F. D-4F also increased the serum levels of pre-β HDL in PyMT mice, which was associated with improved ability of D-4F to stimulate cholesterol efflux from MCF-7 cells. This change could also be associated with a reduction in cholesterol bioavailability for cellular proliferation^30^.

A previous report showed that L-4F associated preferentially with HDL particles after it was injected into mouse circulation and it was cleared rapidly from circulation^31^. The presence of 4F in HDL allowed the transfer of oxidized lipids (majorly oxidized phospholipids) from LDL to HDL and their pre-β HDL-facilitated elimination^19^,^31^. Nevertheless, it has also been demonstrated that 4F preferentially targets the small intestine, and is predominantly transported into the intestinal lumen via a trans-intestinal pathway^32^. Although we cannot rule out the direct effect of D-4F within mammary glands, our results strongly indicate that the D-4F-mediated effects on oxLDL formation counteract the pro-carcinogenic signalling of these modified lipoproteins. However, D-4F treatment did not inhibit the development of hyperplastic lesions at 45 days of age (7.81 ± 1.41 vs. 7.15 ± 0.39 mm^2^ of total lesion area in D-4F- vs. vehicle-treated mice; p = 0.66); this finding indicates that the effects of D-4F are prominent in more advanced stages of the disease.

It should be noted that D-4F-mediated changes in oxLDL levels were not associated with higher HDLc levels. We also found that D-4F downregulated liver *Abca1* expression, although *Apoa1* expression was upregulated (Supplementary Fig. S5). These findings suggest that the D-4F-mediated reduction in HDL mass could be related to a decrease in HDL biosynthesis. However, the injection of an apoA-I mimetic peptide similar to D-4F in C57BL/6J wild-type mice induced a dose-dependent decrease in mature HDL and increased the pre-β HDL levels^20^. This indicates that the peptide may induce the remodelling of mature HDL into pre-β HDL. The reduction of HDLc by apoA-I mimetics has not been reported in other mouse models of dyslipemia and cancer^33^,^34^. However, the HDL-lowering effects of D-4F in our mice could be related to the long period of treatment, since D-4F was not observed to affect HDLc by 45 days of age (1.24 ± 0.12 vs. 1.38 ± 0.03 mM in D-4F- vs. vehicle-treated mice; p = 0.27).

We also examined the effect of increase in the HDL levels on tumour progression by overexpressing hApoA-I. Human apoA-I overexpression did not affect tumour development in this mouse model, even though the oxLDL levels were reduced. However, overexpression of hApoA-I also increased the 27-HC levels in PyMT mice, and this has been reported to promote tumour growth in mouse models of oestrogen-receptor-positive breast cancer^21^,^22^. It should be noted that high-fat- and high-cholesterol-containing diets (which were not used in the present study) also led to an increase in the HDLc levels^35^ and accelerated tumour growth in PyMT mice^36^. HDL is an important carrier of 27-HC^37^ and HDLc-mediated uptake by SR-BI is required for cellular proliferation and breast cancer development^38^. Therefore, although SR-BI was not upregulated in the PyMT-hApoA-I mice, the higher hApoA-I-containing HDL levels together with the lower cholesterol efflux could have promoted 27-HC accumulation in the mammary glands^39^. In human breast cancer tissue, a 50% downregulation of *Cyp7b1* mRNA, which metabolizes 27-HC^40^, was correlated with an increase in 27-HC levels in tumours, presumably due to a delay in catabolism^22^ and worsening of the prognosis^21^. Furthermore, 27-HC may activate LXR, which attenuates the expression of *Cyp7b1*^41^. Consistent with these findings, *Cyp7b1* expression was downregulated in PyMT-hApoA-I mice, which could have enhanced the increase in the mammary gland 27-HC content and counteracted the HDL-mediated benefits on the oxLDL levels. However, D4-F did not affect the mammary gland 27-HC content even though it reduced the serum 27-HC levels; thus, *Cyp7b1* expression seems to be critical for the regulation of tissue 27-HC levels.

As noted previously, the apoA-I mimetic peptide D-4F has been reported to exert anti-tumourigenic effects via its inhibition of LPA in mouse models of ovarian and colon cancer^10^,^16^. However, we did not find any correlation between the serum levels of LPA molecular species and tumour growth in our D-4F-treated PyMT mice. Similar results were observed with the anti-tumourigenic mimetic peptide 6F in a mouse model of ovarian cancer^34^. These results suggest the different potential mechanisms of D-4F-mediated anti-tumour activity. A previous report revealed a potent immunomodulatory role of lipid-free apoA-I in the tumour microenvironment of melanomas via promotion of tumour-associated macrophages (TAMs) with an anti-tumour pro-inflammatory phenotype^11^. However, in our study, treatment with D-4F did not result in increased pro-inflammatory gene expression in the tumourigenic mammary tissue.

Recently, Chattopadhyay *et al.*^42^ reported that feeding mice a concentrate of transgenic tomatoes expressing the apoA-I mimetic peptide 6F decreased the formation of oxidized phospholipids in the jejunum. The decrease in oxidized phospholipids was associated with decreased inflammation in the jejunum and systemically^42^. We did not directly measure the level of the oxidized phospholipids formed in this study. However, our findings with regard to oxLDL are consistent with a similar mode of action.

In summary, we demonstrate here that the apoA-I mimetic peptide D-4F, but not an increase in apoA-I-containing HDL levels, inhibits tumour growth in mice with inherited breast cancer. Our data are consistent with the reported anti-tumourigenic activity of apoA-I mimetics in other type of cancers^10^,^16^,^34^ and, therefore, warrants further study.

## Material and Methods

### Mice and diets

PyMT transgenic mice express high levels of the transforming oncogene polyoma middle T antigen under the control of the mouse mammary tumour virus long terminal repeat promoter, which specifically directs expression in the mammary epithelium^43^. PyMT mice with an FVB/N background was obtained from The Mouse Models of Human Cancers Consortium Repository (National Cancer Institute, Frederick, MD, USA) and backcrossed into the C57BL/6 background for nine generations. These PyMT mice were backcrossed with human apoA-I (hApoA-I) transgenic mice (Jackson Laboratories, Bar Harbor, ME; #003904) with a C57BL/6J background (experiment A) or given the apoA-I mimetic D-4F (Ac-DWFKAFYDKVAEKFKEAF-NH_2_, 10 mg/kg, subcutaneously [s.c.]) or vehicle (saline, s.c.) daily from weaning to 45 days of age, and three times per week until they reached 19 weeks of age (experiment B). A schematic representation of both experiments is shown in Supplementary Fig. S6. In both experiments, the animals were maintained on regular chow diet (A04; Scientific Animal Food & Engineering, Augy, France) containing 3% fat and 0.02% cholesterol. All animals were kept in a temperature-controlled environment (20 °C) with a 12-hour (h) light/dark cycle. Food and water were provided *ad libitum.* Genotyping was performed as indicated on the Jackson Laboratory web site (http://jaxmice.jax.org). The animal protocols used in this study were approved by the Institutional Animal Care and Use Committee of the Institut de Recerca de l’Hospital de la Santa Creu i Sant Pau and the methods were carried out in accordance with the approved guidelines. At the end of the studies, mice were kept on an overnight (o/n) fast, euthanized, and exsanguinated by cardiac puncture. Blood was collected and serum was obtained by centrifugation. Food intake was monitored for 2 days before the animals were euthanized.

### Whole-mount analysis of mammary glands

The right inguinal mammary glands of 45-days-old females were excised, spread onto glass slides, fixed in ethanol/acetic acid (3:1, v/v) for 2 h and stained overnight with carmine alum as previously described^36^. Whole mounts were digitally photographed beside a ruler, and the total area measurements for the hyperplastic lesions were quantified using the Image J software (available at https://imagej.nih.gov/ij/).

### Tumour analyses

The mice from all the experiments were monitored every 3 days for palpable tumours starting at 6 weeks of age. Tumour latency was defined as the time to the development of the first palpable tumour in each mouse. Total tumour burden was determined after all the mammary glands were carefully excised and weighed (mammary gland localization is schematically represented in Supplementary Fig. S7), and the mass of the tumour-bearing mammary glands was determined in 19-week-old mice^36^,^44^. All mammary glands were then frozen in liquid nitrogen, with the exception of the right inguinal mammary glands, which were fixed in 10% neutral-buffered formalin (Sigma-Aldrich, St. Louis, MO), and embedded in paraffin after dehydration. Sections were cut at 5 μm, stained with hematoxylin and eosin and evaluated blindly by an experienced histopathologist. Each section was graded as normal, hyperplasia, adenoma or carcinoma according to the maximum lesion grade, based on previous guidelines^44^,^45^.

### Assessment of the lipid, lipoprotein and apolipoprotein levels

Serum lipid analyses were determined enzymatically using commercial kits adapted to a COBAS c501 autoanalyzer (Roche Diagnostics, Rotkreuz, Switzerland)^35^. HDL cholesterol was measured in apoB-depleted serum obtained after precipitation with phosphotungstic acid and magnesium ions (Roche Diagnostics).The composition of HDL, including total and free cholesterol, phospholipids, and apolipoproteins, was determined in apoB-depleted serum and used to calculate the total HDL mass. The serum human apoA-I levels were determined using nephelometric commercial kits adapted to a COBAS c501 autoanalyzer (Roche Diagnostics). Mouse apoA-I (mApoA-I) was quantified with an ELISA kit, and the wells were coated with a polyclonal rabbit antibody against mApoA-I, as previously reported^46^. The amount of pre-β HDL was quantified by resolving the samples with two-dimensional crossed immunoelectrophoresis, as previously reported^47^. Serum oxLDL was assayed with an ELISA kit (Cloud-Clone, Houston, TX) following the manufacturers’ instructions. The serum LPA levels were determined using high-performance liquid chromatography tandem mass spectrometry (HPLC-MS/MS) as described previously^48^. The concentration of 27-HC from serum and left thoracic mammary gland was measured using HPLC-MS/MS after lipid extraction^49^. HDL (density range 1.063–1.21 g/mL) was isolated from wild-type or hApoA-I transgenic mice by sequential ultracentrifugation at 100,000 g with an analytical fixed-angle rotor (50.3; Beckman Coulter, Fullerton, CA)^18^. The HDL protein concentration was determined using the bicinchoninic acid method (TermoScientific, Rockford, IL). Control human LDL (1.019–1.063 g/mL) was isolated from a pool of normolipemic plasma, dialyzed against phosphate-buffered saline (PBS) by gel filtration chromatography and oxidized with 10 μM of CuSO_4_ for 1 h and 45 min at 37 °C. The apoB concentration was determined using nephelometric commercial kits (Roche Diagnostics).

### Susceptibility to lipoprotein oxidation

The ability of HDL or D-4F to prevent LDL oxidation was evaluated in an assay in which human LDL was oxidized by CuSO_4_ alone in the presence of HDL or the mimetic. Oxidation kinetics were followed by continuous monitoring of the formation of conjugated dienes^50^. After 2.5 μM CuSO_4_ was added to the lipoproteins, continuous monitoring was performed at 234 nm absorbance (BioTek Synergy, Winooski, VT) at 37 °C for 4 h, using a 96-microwell plate for UV detection (Greiner, Hannover, Germany). The lag phase was calculated as described previously^50^. The kinetics of LDL in LDL+HDL incubations was calculated by subtracting the kinetics of HDL incubated without LDL from the total kinetics.

### Cell-culture experiments

The control human oxLDL and HDL from each experimental group were dialyzed against DMEM by gel-filtration chromatography. MCF-7 cells were cultured in DMEM supplemented with 10% foetal bovine serum (FBS) and 100 U/mL penicillin/streptomycin (P/S) (Lonza, Verviers, Belgium) in culture plates, equilibrated o/n in FBS-free medium and then incubated with or without 100 mg/L of oxLDL alone or in combination with 100 mg/L of HDL or 20 mg/L of D-4F mimetic in DMEM supplemented with 5% human lipoprotein-depleted serum (LPDS) and 1% P/S for 48 h. For gene expression analyses, MCF-7 cells (6 × 10^5^ cells/well) were seeded in 12-well culture plates, equilibrated o/n in FBS-free medium and then incubated with 20 mg/L of D-4F in DMEM supplemented with 5% human LPDS for 48 h.

### Cell viability

Cell viability was assayed in MCF-7 cells seeded in 96-well culture plates at 10^4^ cells/well and treated as previously described using the Vybrant MTT cell proliferation assay kit (Thermo Fischer Scientific, Waltham, MA).

### Cell migration

MCF-7 cells (5 × 10^5^ cells/well) were seeded in 6-well culture plates and equilibrated o/n in FBS-free medium. The wound area was created with a pipette tip, and 20 mg/L of D-4F was added to the wound area. The wound area was photographed at 0 and 16 h and quantified using the Image J software.

### *In vitro* cholesterol efflux

*In vitro* cholesterol efflux from MCF-7 cells was analysed by adapting the previously described methodology^51^. Briefly, MCF-7 cells (2 × 10^5^ cells/well seeded in 6-well culture plates) were labelled for 60 h with [1α,2α(n)-^3^H]cholesterol (GE Healthcare, 1 μCi per well). The labelled cells were equilibrated o/n in 0.2% bovine serum albumin (BSA) and 0.3 mM of cyclic adenosine monophosphate (cAMP) (Sigma-Aldrich) and then incubated for 4 h at 37 °C with 20 mg/L of hApoA-I (Sigma-Aldrich) or D-4F. Radioactivity was then measured in both media and cells and the percentage of cholesterol efflux were determined.

### Quantitative real-time PCR

Total RNA from the right cervical mammary gland and liver was isolated using the TRIzol^®^ reagent (Invitrogen, Carlsbad, CA) following the manufacturer’s protocol and purified using the RNeasy Plus Mini Kit (Qiagen, Hilden, Germany). Total RNA from MCF-7 cells was isolated using the RNeasy Plus Mini Kit (Qiageny). cDNA was generated using Oligo (dT)_23_ and a mixture of dNTPs (Sigma Aldrich), and M-MLV Reverse Transcriptase, RNase H Minus, and Point Mutant (Promega, Madison,WI). cDNA was subjected to quantitative real-time PCR amplification using Taqman Master Mix (Applied Biosystems, Foster City, CA). Specific mouse Taqman probes (Applied Biosystems) were used for *Abca1* (Mm00442646_m1), *Abcg1* (Mm00437390_m1), *Apoa1* (Mm00437569_m1), *Cyp27a1* (Mm00470430_m1), *Cyp7b1* (Mm00484157_m1), *Lcat* (Mm00500505_m1), *Nr1h3* (Mm00443451_m1), *Pltp* (Mm00448202_m1), *Scarb1* (Mm00450236_m1) and *Gapdh* (Mm99999915_g1) (reference gene) in mouse tissue gene expression analysis; and specific human Taqman probes (Applied Biosystems) were used for *NR1H3* (Hs00172885_m1), *ABCA1* (Hs01059122_m1), *ABCG1* (Hs00245154_m1), *SCARB1* (Hs00969827_m1), *CYP27A1* (Hs01026016_m1), *CYP7B1* (Hs00191385_m1) and *ACTB* (Hs99999903_m1) (reference gene) in MCF-7 gene expression analysis. Reactions were run on a CFX96^TM^ Real-Time System (Bio-Rad, Hercules, CA) according to the manufacturer’s instructions. cDNA was also subjected to quantitative real-time PCR amplification using the SYBR green master mix (Applied Biosystems) and specifically designed primers (Supplemental Table S1) for markers of macrophage activation. Reactions were run on an ABI PRISM 7900 HT (Applied Biosystems) according to the manufacturer’s instructions. The relative mRNA expression levels were calculated using the ΔΔCt method.

### Western blot analysis

Proteins from the right cervical mammary gland were also isolated using the TRIzol^®^ reagent (Invitrogen) following the manufacturer’s protocol, fractionated on a reducing 10% SDS-PAGE gel, transferred onto a nitrocellulose membrane and then immunoblotted with polyclonal rabbit antimouse SR-BI antibody (Novus Biologicals, Littleton, CO) as previously described^52^.

### Statistical analysis

The log-rank and Gehan-Wilcoxon tests were used to compare latency curves. The influence of D-4F on mammary gland weight in the different localizations and the interaction between these two variables were analysed by two-way ANOVA. When data were normally distributed, the student’s unpaired t-test was used to compare differences between groups; the nonparametric Mann–Whitney test was used for data that did not follow Gaussian distribution. The effects of HDL or D-4F on cell viability and LDL oxidation assays were analyzed by one-way ANOVA. The relationship between lipoprotein variables and tumour burden was determined using Pearson’s correlation test. The GraphPad Prism 6.0 software (GraphPad, San Diego, CA) was used to perform all statistical analyses. A *p* value of ≤0.05 was considered to indicate statistical significance.

## Additional Information

**How to cite this article**: Cedó, L. *et al.* ApoA-I mimetic administration, but not increased apoA-I-containing HDL, inhibits tumour growth in a mouse model of inherited breast cancer. *Sci. Rep.* **6**, 36387; doi: 10.1038/srep36387 (2016).

## Supplementary Material

Supplementary Information

## Figures and Tables

**Table 1:** Body weight, food intake and serum parameters in PyMT and PyMT-hApoA-I mice.

	PyMT	PyMT-hApoA-I	p
Body weight (g)	22.95 ± 0.55	23.67 ± 0.58	0.38
Food intake (g/day)	3.41 ± 0.33	3.80 ± 0.16	0.29
Total serum cholesterol (mmol/L)	2.05 ± 0.10	3.72 ± 0.21	<0.0001
HDL cholesterol (mmol/L)	1.80 ± 0.14	3.56 ± 0.25	<0.0001
HDL mass (g/L)	3.08 ± 0.20	8.12 ± 0.34	<0.001
hApoA-I (g/L)	ND	2.99 ± 0.17	
mApoA-I (g/L)	1.04 ± 0.10	0.66 ± 0.09	<0.05
pre-β HDL levels (% of mApoA-I)	0.51 ± 0.51	1.78 ± 0.81	0.21

Values represent the mean ± SEM for 8–10 mice/group. ND, non-detected; hApoA-I, human apoA-I; mApoA-I, mouse apoA-I. Statistical significance was considered when p ≤ 0.05 vs. the PyMT group.

**Table 2:** Body weight, food intake and serum parameters in PyMT mice treated with the apoA-I mimetic D-4F or the vehicle.

	Vehicle	D-4F	p
Body weight (g)	22.45 ± 0.85	19.99 ± 0.25	<0.01
Food intake (g/day)	3.90 ± 0.32	4.01 ± 0.17	0.76
Total serum cholesterol (mmol/L)	2.04 ± 0.13	1.80 ± 0.09	0.15
HDL cholesterol (mmol/L)	1.59 ± 0.12	1.14 ± 0.08	<0.01
HDL mass (g/L)	2.93 ± 0.18	2.46 ± 0.14	<0.05
mApoA-I (g/L)	0.80 ± 0.09	0.79 ± 0.13	0.52
pre-β HDL levels (% of mApoA-I)	0.51 ± 0.51	3.26 ± 0.94	<0.05

Values represent the mean ± SEM for 8 mice/group. mApoA-I, mouse apoA-I. Statistical significance was considered when p ≤ 0.05 vs. the vehicle.
