# Supplementary material for: ApoA-I mimetic administration, but not increased apoA-I-containing HDL, inhibits tumour growth in a mouse model of inherited breast cancer
Source: Sci Rep. 2016 Nov 3;6:36387. doi: 10.1038/srep36387 (PMC5093413; doi:10.1038/srep36387)
Supplement: Supplementary Information [file srep36387-s1.doc]

**ApoA-I mimetic administration, but not increased apoA-I-containing HDL, inhibits tumour growth in a mouse model of inherited breast cancer**

Lídia Cedó, Annabel García-León, Lucía Baila-Rueda, David Santos, Victor Grijalva, Melanie Raquel Martínez-Cignoni, José M. Carbó, Jari Metso, Laura López-Vilaró, Antonio Zorzano, Annabel F. Valledor, Ana Cenarro, Matti Jauhiainen, Enrique Lerma, Alan M. Fogelman, Srinivasa T. Reddy, Joan Carles Escolà-Gil, Francisco Blanco-Vaca

**
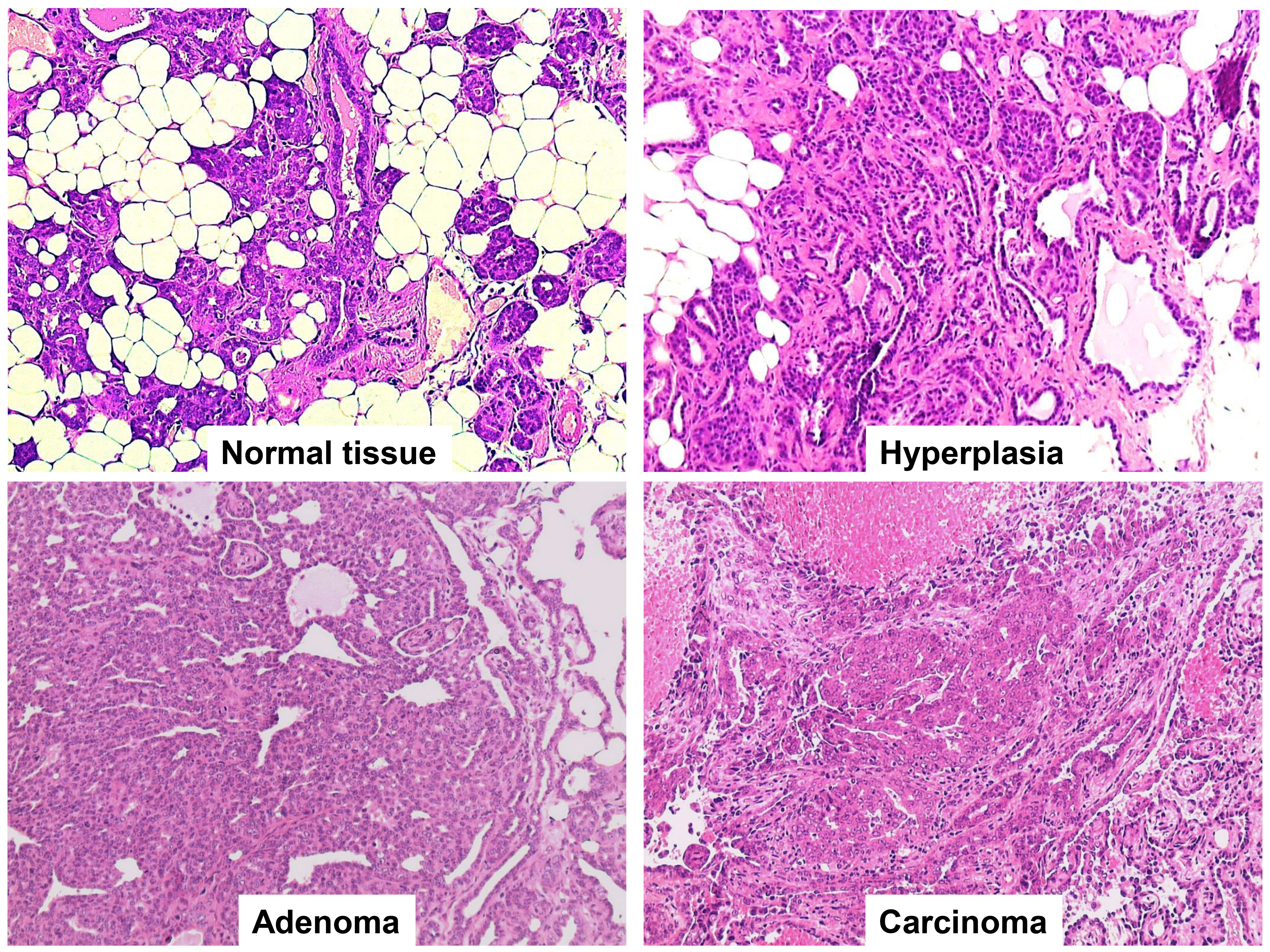
**

**Supplementary Figure S1.** Representative images of each histopathologic stage in mammary gland lesion grade study. Original magnification ×10.

**
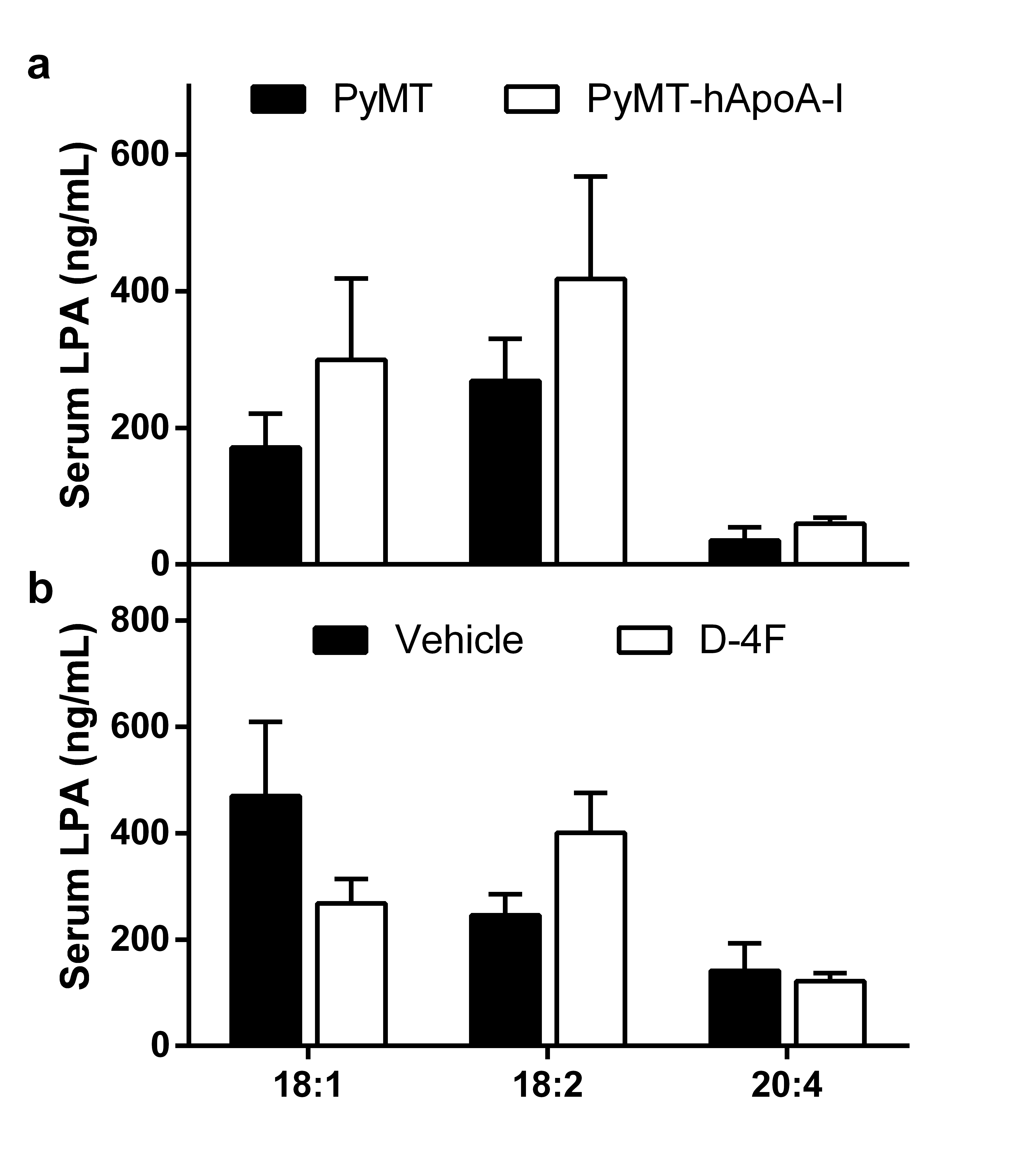
**

**Supplementary Figure S2.** Serum LPA levels in (a) PyMT and PyMT-hApoA-I mice and in (b) PyMT mice treated with 10 mg/kg of D-4F or the vehicle. The values represent the mean ± SEM for 5 individual animals per group.

**
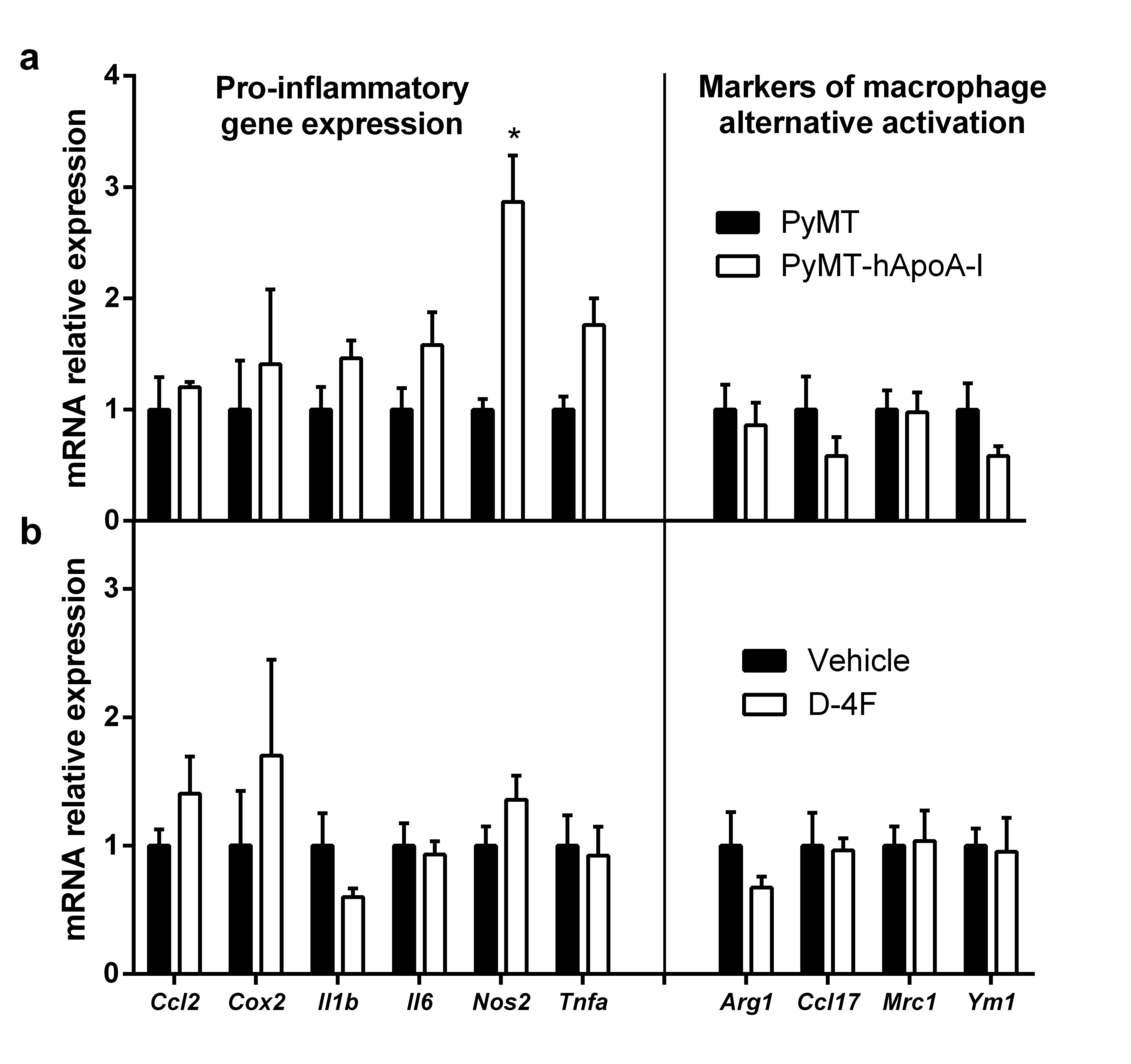
**

**Supplementary Figure S3.** Analysis of the expression of prototypical macrophage activation markers in right cervical mammary gland from (a) PyMT and PyMT-hApoA-I mice and from (b) PyMT mice treated with 10 mg/kg of D-4F or the vehicle. The signal of the PyMT or vehicle group was set at a normalized value of 1 AU. *Rpl13a* was used as the internal control. The values represent the mean ± SEM for 5-7 individual animals per group, and * indicates p ≤ 0.05 vs. the PyMT or vehicle group, respectively.


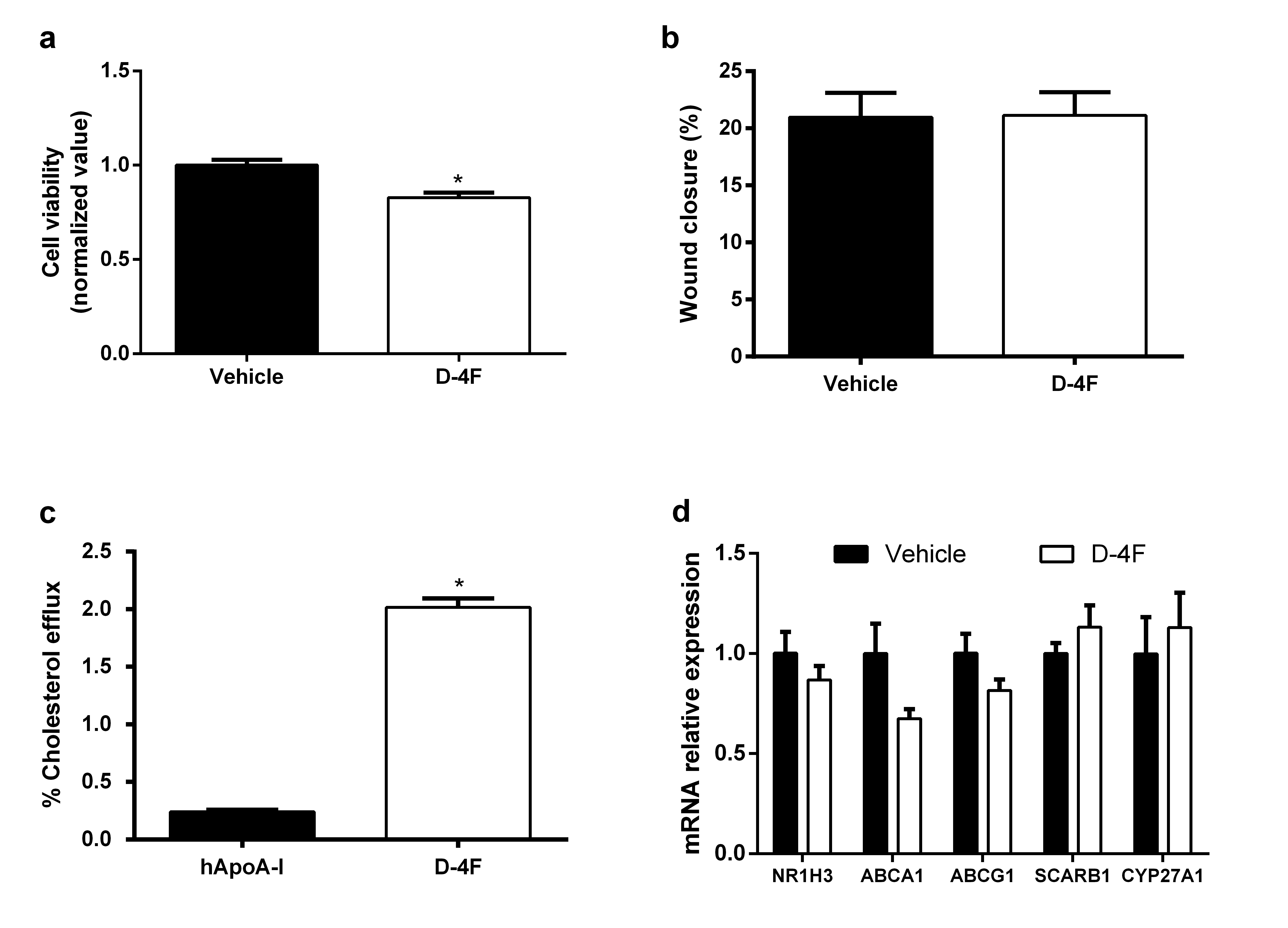


**Supplementary Figure S4.** Effects of D-4F on MCF-7 cells. (a) Cell viability of MCF-7 cells treated with 20 mg/L of D-4F. The signal of the control group was set at a normalized value of 1 AU. The values represent the mean ± SEM of 6 individual replicates (b) *In vitro* migration of MCF-7 cells to the closure, treated with 20 mg/L of D-4F for 16 hours. The values represent the mean ± SEM of 3 independent experiments (5 individual replicates per experiment and group) (c) *In vitro* cholesterol efflux from [^3^H]cholesterol-labelled MCF-7 cells to 20 mg/L of human apoA-I or D-4F at 4 hours. The values represent the mean ± SEM of 8-10 individual replicates (d) Gene expression in MCF-7 cells treated with 20 mg/L of D-4F. The signal of the vehicle group was set at a normalized value of 1 AU. *ACTB* was used as the internal control. The values represent the mean ± SEM and 6 independent experiments. * indicates p ≤ 0.05 vs. the vehicle group.


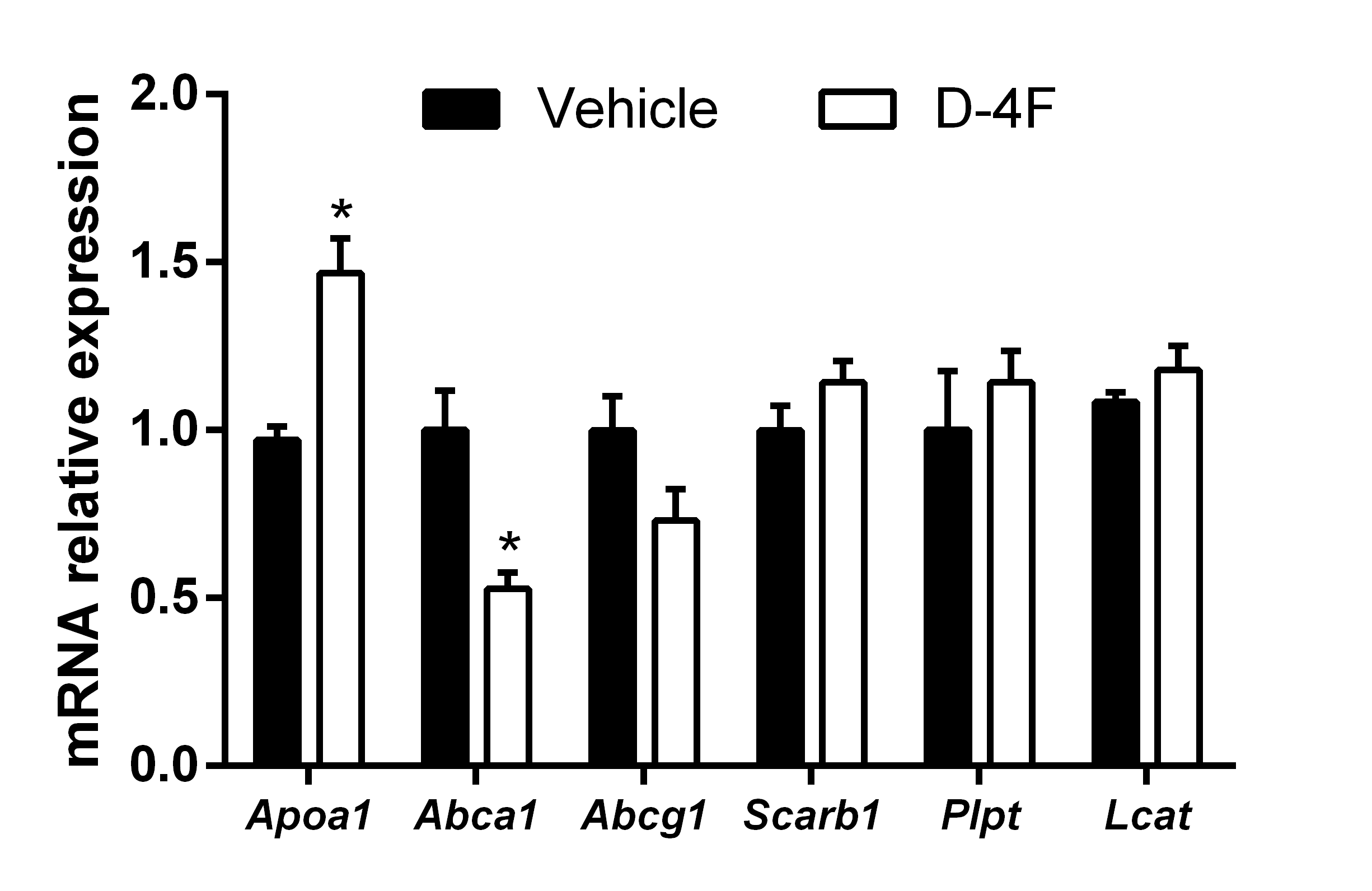


**Supplementary Figure S5.** Effects of D-4F treatment on liver gene expression in PyMT mice. The signal of the vehicle group was set at a normalized value of 1 arbitrary unit (AU). *Gapdh* was used as the internal control. The values represent the mean ± SEM for 6 individual animals per group. * indicates p ≤ 0.05 vs. the vehicle group.


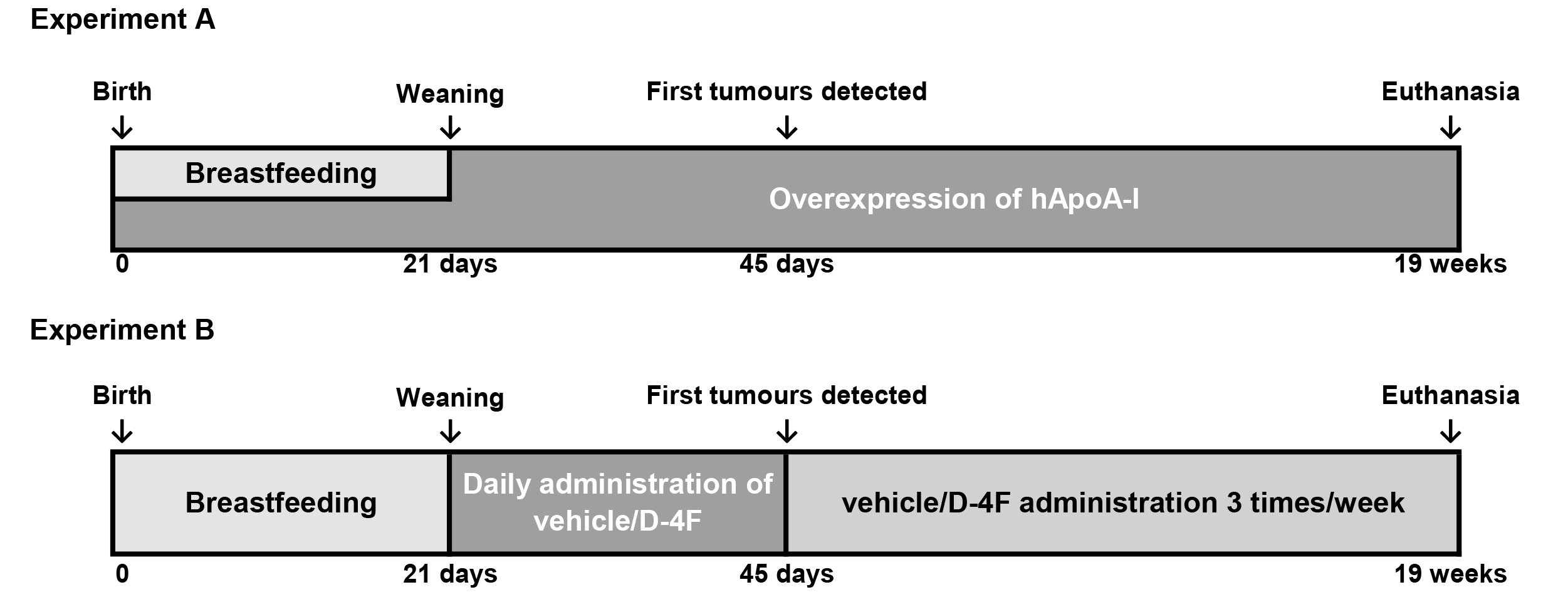


**Supplementary Figure S6.** Schematic representation of Experiment A and B.

**
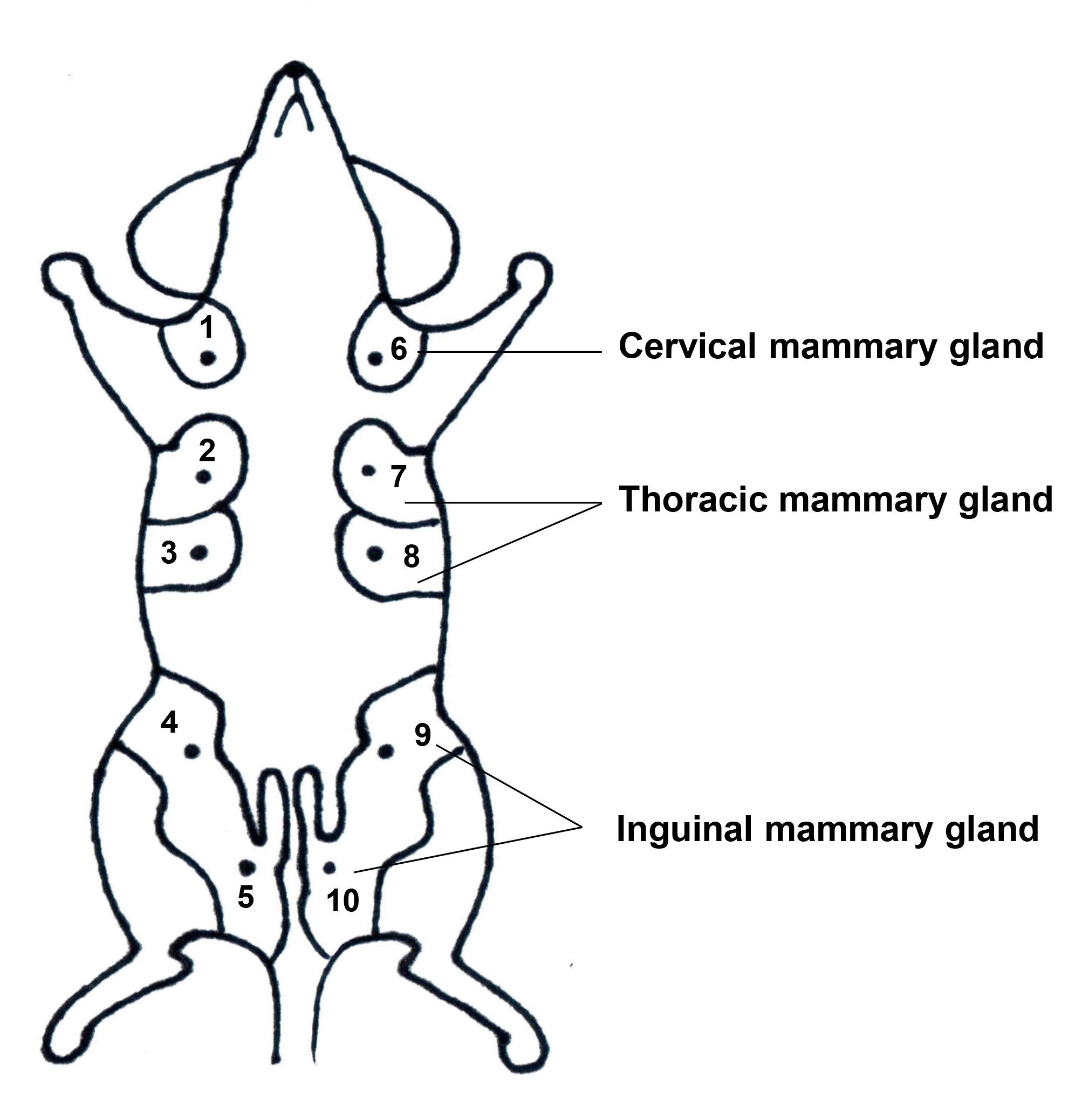
**

**Supplementary Figure S7.** Schematic representation of mammary gland localization in mice.

**Supplementary Table S1:** Mice-specific primer sequences for M1/M2 markers.

| **Gene** | **Primer sequence** |
| --- | --- |
| *Arg1* | F: 5’ TTGCGAGACGTAGACCCTGG 3’  R: 5’ CAAAGCTCAGGTGAATCGGC 3’ |
| *Ccl2* | F: 5’ AGGTCCCTGTCATGCTTCTG 3’  R: 5’ GCTGCTGGTGATCCTCTTGT 3’ |
| *Ccl17* | F: 5’ TGCTTCTGGGGACTTTTCTG 3’  R: 5’ CATCCCTGGAACACTCCACT 3’ |
| *Cox2* | F: 5’ ATTCTTTGCCCAGCACTTCA 3’  R: 5’ GGGATACACCTCTCCACCAA 3’ |
| *Il1b* | F: 5’ TGGGCCTCAAAGGAAAGAAT 3’  R: 5’ CAGGCTTGTGCTCTGCTTGT 3’ |
| *Il6* | F: 5’ CCAGAGATACAAAGAAATGATGG 3’  R: 5’ ACTCCAGAAGACCAGAGGAAAT 3’ |
| *Mrc1* | F: 5’ GGTTCACCTGGAGTGATGGTTC 3’  R: 5’ GTGGATTGTCTTGTGGAGCAGG 3’ |
| *Nos2* | F: 5’ GCCACCAACAATGGCAACA 3’  R: 5’ CGTACCGGATGAGCTGTGAATT 3’ |
| *Rpl13a (reference)* | F: 5’ GAGGAGGGAGATGTGATGGA 3’  R: 5’ ACTCTGGGACTCCATGTTGG 3’ |
| *Tnfa* | F: 5’ CCAGACCCTCACACTCAGATC 3’  R: 5’ CACTTGGTGGTTTGCTACGAC 3’ |
| *Ym1* | F: 5’ TGTTCTGGTGAAGGAAATGCG 3’  R: 5’ CGTCAATGATTCCTGCTCCTGT 3’ |
